# A systematic review of instruments for the analysis of national-level physical activity and sedentary behaviour policies

**DOI:** 10.1186/s12961-019-0492-4

**Published:** 2019-11-13

**Authors:** Bojana Klepac Pogrmilovic, Grant O’Sullivan, Karen Milton, Stuart J. H. Biddle, Zeljko Pedisic

**Affiliations:** 1 Institute for Health and Sport, Victoria University, Ballarat Road, Footscray, Melbourne, VIC 3001 Australia; 2 Norwich Medical School, University of East Anglia, Norwich Research Park, Norwich, United Kingdom; 3 Centre for Health, Informatics, and Economic Research, Institute for Resilient Regions, University of Southern Queensland, Springfield, Australia

**Keywords:** Physical activity, sedentary behaviour, national policy, policy analysis, instrument, tool, framework

## Abstract

**Background:**

This systematic review aimed to identify and critically assess available instruments for the analysis of national-level physical activity (PA) and sedentary behaviour (SB) policies and provide recommendations for their future use.

**Methods:**

We conducted a systematic search of academic and grey literature through six bibliographic databases, Google and the websites of three international organisations for PA promotion to identify instruments that are used or that may be used for national-level PA/SB policy analysis. In order to describe and categorise the identified instruments, we used the Comprehensive Analysis of Policy on Physical Activity framework. This framework specifies the elements of a comprehensive analysis of PA/SB policies through the following categories: purpose, level, policy sector, type of policy, stages of policy cycle and scope of analysis.

**Results:**

Out of 22,071 screened items, 26 publications describing 16 instruments met the selection criteria. All the instruments can be used for analysing PA policy, whilst only two include questions about SB policy. None of the instruments allow for the analysis of all the relevant components of national PA/SB policy. Some important elements of PA policy analysis, such as the tourism and research sectors, the agenda-setting and endorsement/legitimisation stages, and the effects of policy, are addressed by only a few instruments. Moreover, none of the instruments address unwritten formal statements, informal policies, and the termination and succession stages of the policy cycle.

**Conclusion:**

Designing new instruments or adapting existing ones is needed to allow for a more thorough analysis of national PA and SB policies. Given that policy analysis covering all important components of PA/SB policy may be extremely time-consuming, a way forward might be to develop a set of complementary instruments, with each tool collecting detailed information about a specific component.

## Background

In 2008, it was estimated that 1 in 10 deaths worldwide were attributable to insufficient physical activity (PA) [1]. If rates of physical inactivity were to be reduced by just 10% to 20%, between half a million to more than a million lives could be saved each year [1]. It was estimated that, from 2002 to 2011, sedentary behaviour (SB) was responsible for 3.8% of all deaths [2]. Physical inactivity and SB are not just key contributors to global mortality but there is also substantial economic burden to national healthcare systems worldwide associated with these behaviours. Estimates suggest that the lack of PA costs countries around the world over 50 billion dollars a year, of which almost 70% is paid by the public sector [3].

Both SB and insufficient PA are among the key risk factors for non-communicable diseases (NCDs) such as type 2 diabetes, cancer and cardiovascular disease. NCDs are responsible for the deaths of almost 40 million people per year, which is approximately 70% of the overall global mortality [4]. Furthermore, low levels of PA and high levels of SB are also associated with negative mental health outcomes [5, 6].

National governments are crucial players in achieving positive changes in population health [7]. Governments are, in cooperation with other public health stakeholders, responsible for creating environments that empower individuals to make health-enhancing decisions [7]. One of the essential determinants of active living is the policy environment [8], and the development and implementation of national policies may contribute to the creation of supportive environments for people to engage in physically active lifestyles [9, 10]. The recent Global Action Plan on Physical Activity 2018–2030, issued by WHO, recommends 20 policy actions that produce multiple social, economic and health benefits, and are applicable to different national contexts [10]. Typical examples of standalone PA policies are national PA action plans (e.g. ‘Get Ireland Active!’ – the national physical activity plan for Ireland [11]) and national PA strategies (e.g. ‘Everybody active, every day’ – an evidence-based approach to physical activity by Public Health England [12]). PA and SB policies are also often included in national obesity prevention strategies (e.g. the Mexican National Strategy for the Prevention and Control of Overweight, Obesity and Diabetes [13]), NCD prevention strategies (e.g. National Multisectoral Strategic Plan for Prevention and Control of Non-Communicable Diseases in Namibia 2017/18–2021/22 [14]), and public health strategies (e.g. ‘Healthy throughout Life’ – the targets and strategies for public health policy of the Government of Denmark, 2002–2010 [15]).

Progress regarding the development of national PA policies has been made in most countries [16]. However, with policy implementation generally being poor, countries are urged to take bold initiatives to address this issue [16]. PA and SB policy analysis can help tackle these challenges through raising awareness of the current opportunities and gaps, promoting important cross-sectoral and cross-level debates [17], providing a platform to improve public policy-making related to PA/SB, contributing to meeting various health objectives [18]. and assisting policy-makers in making better informed decisions [19].

Policy analysis, defined as “*any form of policy-relevant research*” [20], encompasses the use of various instruments, tools and techniques to study established policies as well as their development and consequences [21]. It is a valuable practice for continuous improvement of policies, and it has been developing for almost 70 years [22, 23]. Health policy analysis has a central role in fostering successful health promotion reforms [24]. There is no consensus on how to perform a policy analysis and which method is best [25]. A plethora of instruments, tools and techniques are available for policy analysis in general [23, 26–29], health policy analysis [21, 24, 30], and specific areas within health policy such as chronic illness [31] or obesity policies [32]. Given that contexts and research questions relevant for policy analysis in different areas may greatly differ, not all policy analysis instruments are universally applicable. Several instruments have, therefore, been developed specifically for the analysis of PA and SB policies [33, 34]. The Comprehensive Analysis of Policy on Physical Activity (CAPPA) framework [35] defines 38 elements of a comprehensive analysis of PA and SB policies, through the following 6 categories: ‘purpose’, which includes 2 elements – auditing and assessment of policies; ‘level’, which includes 5 elements – international, national, subnational, local and institutional policies; ‘policy sector’, which includes 11 elements – health, sport, recreation and leisure, education, transport, environment, urban/rural planning and design, tourism, work and employment, public finance, and research; ‘type of policy’, which includes 5 elements – formal written policies, unwritten formal statements, written standards and guidelines, formal procedures and informal policies; ‘stage of the policy cycle’, which includes 8 elements – agenda-setting, formulation, endorsement/legitimisation, implementation, evaluation, maintenance, termination and succession of policy; and ‘scope of analysis’, which includes 7 elements – availability, context, processes, actors, political will, content and effects. The CAPPA framework also provides definitions and key rationales underpinning each category and element of the framework [35]. PA and SB are co-dependent behaviours [36] and the contexts of PA and SB policies are very similar [35]. Owing to these facts, PA and SB policies are very often studied within a single study. A recent review found only 1 study that analysed SB policies independently of PA policies [25]. It was therefore suggested that the CAPPA framework can be used to guide research on SB policies.

Research on PA policies is growing and is much more developed than it was a few years ago [25]. Although SB policy research is still in its infancy, there has been some progress in recent years [25]. Klepac Pogrmilovic et al. [25] found that various definitions were used to conceptualise PA/SB policy as well as various methodological approaches and instruments to perform PA policy analysis. This lack of standardisation may be desirable in young research fields, as it puts less constraints on methodological approaches, and therefore allows empirical evaluation of different methodologies. However, it may also lead to a vague conceptualisation of research questions and can hinder cross-study and inter-policy comparability [25].

The scope and quality of policy analysis and comparability of findings across studies will largely be determined by the quality, comprehensiveness and uniformity of instruments used to perform the analyses. No previous systematic review has summarised information about the instruments used for the analysis of national policies related to PA and/or SB. Therefore, the aim of this systematic literature review was to identify and critically assess available instruments for the analysis of national-level PA/SB policies and provide recommendations for their future use. We aimed to assess the purpose and scope of each instrument, the sectors and stages of the policy cycle they refer to, and the types of policy that they cover.

## Methods

### Search strategy

**Fig. 1:**
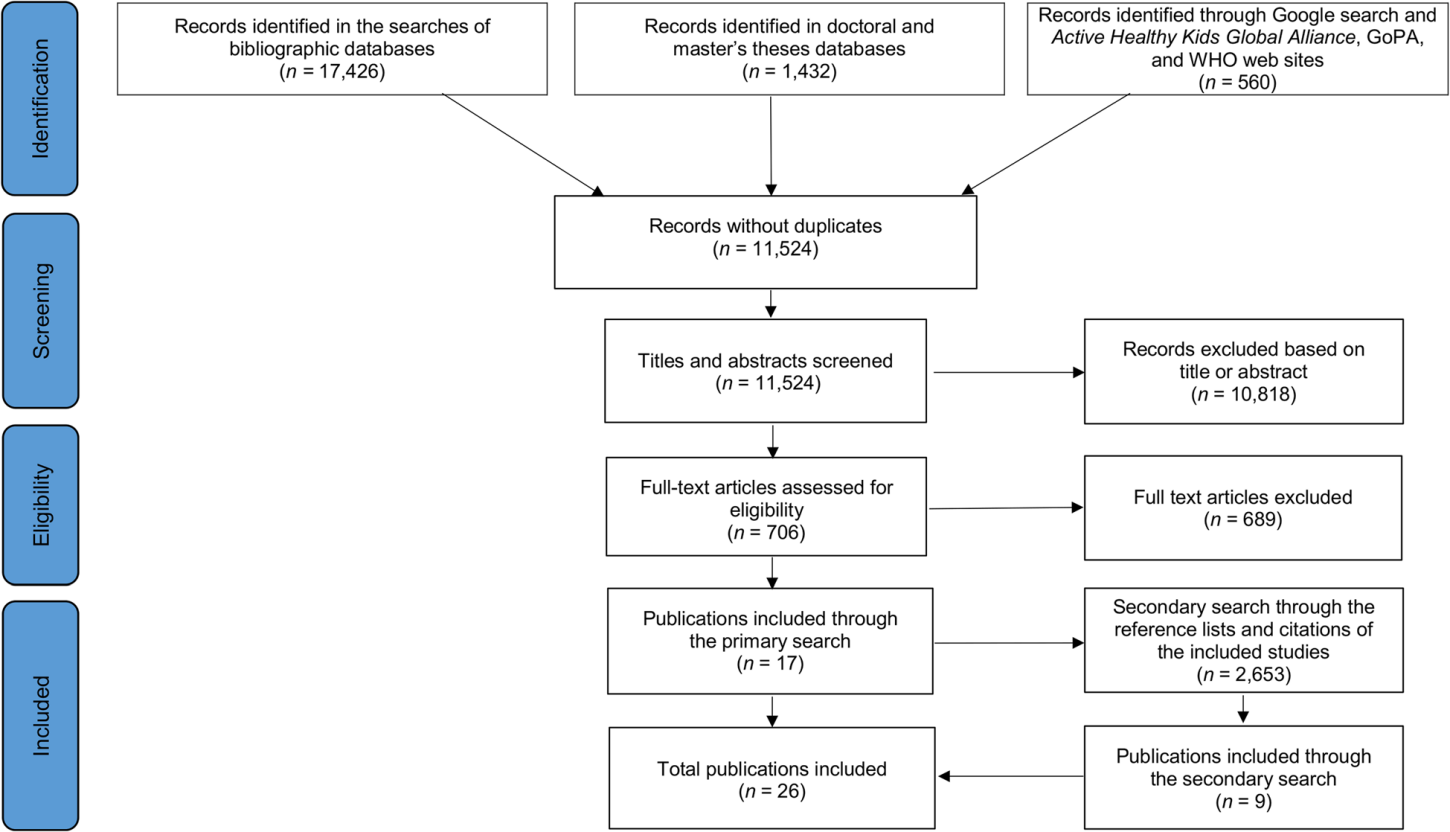
Flow diagram of the search and study selection process

The primary search was conducted in six databases, namely Scopus, SPORTDiscus, PubMed/MEDLINE, Web of Science (including Science Citation Index Expanded, Arts & Humanities Citation Index, Conference Proceedings Citation Index – Science, Social Sciences Citation Index and Conference Proceedings Citation Index – Social Science & Humanities), Networked Digital Library of Theses and Dissertations, and Open Access Theses and Dissertations. The search was conducted through titles, abstracts and keywords using the entries *‘*physical inactivity’, ‘physical activity‘, ‘sitting’ and ‘sedentar*’, and combining them with the terms ‘policy’ and ‘policies’. A full search syntax is available in Additional file 1. The secondary search was performed through (1) the reference lists of all included publications, (2) citations of the included publications identified by Google Scholar and (3) the authors’ own archives. Additional searches were conducted in Google and on the websites of WHO and two large international PA promotion networks – the Active Healthy Kids Global Alliance and the Global Observatory for Physical Activity (GoPA!). We conducted a three-stage screening process that included (1) automatic and manual exclusion of duplicates, (2) manual screening of titles and abstracts, and (3) assessment of eligibility based on full texts. The study selection was completed independently by two authors (BKP and GO) in July 2017. Discrepancies between the study selections were resolved in a discussion with the third author (ZP). If perfect agreement between the three authors had not been reached in the discussion, the final decision was made based on a majority vote. A flow diagram of the search and study selection process is available in Fig. 1.

### Study selection and inclusion criteria

In this review, we considered the term ‘policy analysis’ as a synonym for the terms ‘assessment’, ‘audit’, ‘evaluation’ and ‘review’ of policy. We relied on the definition of PA policy analysis from the CAPPA framework, a conceptual inventory of components for a comprehensive analysis of PA policies, which can be used to guide the selection of existing instruments for policy analysis or the development of new ones [35]. It defines PA policy analysis as “*any kind of policy-relevant research that audits or assesses one or more aspects of PA policy*” [35]. Although developed primarily to guide the analysis of PA policies, the CAPPA framework can also be used in SB policy research [35].

By instruments, we considered sets of criteria and measurement tools that can be used for any aspect of PA/SB policy analysis. By a ‘set of criteria’, we considered a collection of principles that may serve as a guide for policy analysis. These sets of criteria do not usually include specific questions that may directly be used for policy analysis. By contrast, ‘measurement tools’ contain specific questions that may be used in various types of research related to PA/SB policies.

To be included in the review, a publication had to meet the following two criteria:

1. The publication includes an original description of an instrument that has been used or that may be used for national-level PA/SB policy analysis;
2. The abstract and/or the full-text of the publication is available in English.

### Data extraction and coding

The following data were extracted for every identified PA/SB policy analysis instrument: (1) whether it addresses PA policy, SB policy or both; (2) whether its purpose is auditing or assessment of policies; (3) what sectors of policy it covers; (4) what types of policies it covers; (5) what stages of the policy cycle it addresses; and (6) the scope of the policy analysis that can be done using the instrument.

To describe and categorise the identified instruments, we relied on the CAPPA framework [35]. We relied on all categories and elements presented in the CAPPA framework with the exception of a policy level category, because this review focused on national-level policies only.

The data extraction and coding were independently conducted by two authors (BKP and ZP). Disagreements between the authors were resolved by a discussion between all authors. Detailed data extraction is available in Table 1.

**Table 1:** Instruments for the analysis of physical activity and/or sedentary behaviour policies and their characteristics

Instrument	Author(s) and publication	Characteristics					
		Includes items on PA, SB or both	Purpose of analysis	Policy sector	Type of policy	Stage of policy cycle	Scope of analysis
Policy principles for the promotion of healthy diets and physical activity	- WHO, 2003 [37]	PA	Auditing Assessment	None	Formal written policies	Formulation Implementation Evaluation	Processes Actors Content
Criteria for successful policy and action plans on physical activity	- Bull et al., 2004 [38] - Bull et al., 2004 [39] - Schöppe et al., 2004 [40]	PA	Auditing Assessment	None	Formal written policies Formal procedures	Formulation Implementation Evaluation	Context Processes Actors Content
A comprehensive physical activity policy framework	- Shephard et al., 2004 [41]	PA	Auditing Assessment	Education Health Sport Recreation and leisure Transport Urban/rural planning and design Work and employment	Formal written policies Formal procedures	Agenda-setting Formulation Implementation Evaluation	Availability Context Processes Actors Political will Content
Elements of national policy documents	- Branca et al., 2007 [42]	PA	Auditing Assessment	Education Health Transport Urban/rural planning and design Work and employment Public finance Research	Formal written policies	Formulation Implementation Evaluation	Context Actors Content
Key principles that should guide member states in the development of national physical activity strategies	- WHO, 2007 [43]	PA	Auditing Assessment	None	Formal written policies Written standards Formal procedures	None	Availability Context Actors Content Effects
Important elements of successful physical activity policies and plans	- WHO, 2007 [44]	PA	Auditing Assessment	None	Formal written policies Written standards	Implementation Evaluation	Context Processes Actors Political will Content
HARDWIRED criteria for successful national physical activity policy	- Bellew et al., 2008 [45]	PA	Auditing Assessment	None	Formal written policies Written standards Formal procedures	Formulation Implementation Evaluation	Context Processes Actors Political will Content
Eight aspects identified as being relevant for effective physical activity policies	- Daugbjerg et al., 2009 [46]	PA	Auditing	None	Formal written policies Formal procedures	Formulation Endorsement/legitimisation Implementation Evaluation	Actors Content
A graphical, computer-based decision-support tool to help decision makers evaluate policy options relating to physical activity	- Yancey et al., 2010 [47]	PA	Assessment	None	None	Formulation Implementation	Political will Context Effects
Analysis of Determinants of Policy Impact (ADEPT) model	- Rütten et al., 2010 [48] - Rütten et al., 2012 [49]	PA	Auditing Assessment	None	Formal written policies Formal procedures	Formulation Implementation Evaluation	Context Processes Actors Political will Content Effects
Categories for the content analysis of policies	- WHO, 2011 [50] - Christiansen et al., 2014 [51]	PA	Auditing	None	Formal written policies	Formulation Implementation Evaluation Maintenance	Actors Content
HEPA PAT	- Bull et al., 2014 [17] - Bull et al., 2014 [52] - Bull et al., 2014 [53] - Bull et al., 2014 [54] - Bull et al., 2015 [33]	PA and SB	Auditing Assessment	Education Environment Health Sport Recreation and leisure Tourism Transport Urban/rural planning and design Work and employment Public finance Research	Formal written policies Written standards Formal procedures	Formulation Implementation Evaluation Maintenance	Availability Context Processes Actors Political will Content
Government strategies and investments indicator for the Active Healthy Kids report cards	- Tremblay et al., 2014 [55]	PA	Assessment	Public finance	None	Agenda-setting Formulation Implementation Evaluation	Context Actors Political will
Questionnaire on the monitoring framework for the implementation of policies to promote health-enhancing physical activity in the EU and WHO European Region 2015	- WHO, 2015 [56] - European Physical Activity Focal Points Network, 2015 [57]	PA	Auditing	Education Environment Health Sport Recreation and leisure Transport Urban/rural planning and design Work and employment Public finance	Formal written policies Written standards Formal procedures	Implementation	Availability Context Actors Content
Surveillance and policy status indicators for GoPA! country cards	- Ramirez Varela et al., 2016 [58] - Ramirez Varela et al., 2017 [59]	PA	Auditing	None	Formal written policies Formal procedures	None	Availability
GoPA! Policy Inventory version 1.0 (July 2017)	- Global Observatory for Physical Activity, 2017 [34]	PA and SB	Auditing	Education Environment Health Sport Recreation and leisure Transport Urban/rural planning and design	Formal written policies Written standards Formal procedures	Maintenance	Availability Context Actors Content

*GoPA!* Global Observatory for Physical Activity, *HEPA PAT* Health-enhancing physical activity policy audit tool, *PA* physical activity, *SB* sedentary behaviour

## Results

The primary search identified 19,418 records, leaving 11,524 after the removal of duplicates. Following title and abstract screening, 10,818 documents were excluded. Full-texts of the remaining 706 documents were reviewed, and 17 of them were deemed eligible. In the secondary search, we identified a further 2653 documents, 9 of which met the inclusion criteria, providing a total of 26 publications for inclusion (Fig. 1). These 26 documents (12 journal articles [17, 38, 41, 45–49, 51, 52, 55, 59], 11 reports [37, 39, 40, 42–44, 50, 53, 54, 56, 58], 2 published questionnaires [33, 57] and 1 unpublished questionnaire [34]) describe 16 instruments. The identified instruments and their assessments against the CAPPA framework are presented in Table 1. A description of included publications and all instruments is available in Additional file 2. Ten included instruments (described in 13 documents) are sets of criteria [37–46, 50, 51, 55]. To help readers understand how these sets of criteria may be used to collect data about PA/SB policy, we developed sample questions based on the items of 1 set of criteria [37] (Additional file 3). Furthermore, the remaining 6 included instruments (described in 13 documents) are measurement tools [17, 33, 34, 47–49, 52–54, 56–59]. All included publications were issued from 2003 to 2017. Eight studies were funded by the European Union (EU) and/or by WHO.

Only 2 included instruments refer to both SB and PA policies [33, 34]. All other instruments refer to PA policies only. The number of items in the included instruments ranges from 2 to 28 (mode = 8). The included instruments differ greatly in terms of their content and structure. Nevertheless, items about some elements of PA policy emerge repeatedly across multiple instruments. Further, 81% (*n* = 13) of instruments contain items about focus of policy on specific target groups, funding and available resources, and leadership and coordination, and 75% (*n* = 12) of instruments address the importance of integration of PA policy in different sectors and settings. Evaluation of policies and surveillance/monitoring of PA/SB are addressed in 69% (*n* = 11) of the instruments. Setting specific goals for PA promotion is mentioned in 56% (*n* = 9) of the instruments, whilst the importance of involving different stakeholders in PA policy is addressed in 50% (*n* = 8) of the instruments. The significance of political support and the existence of PA guidelines as important parts of a successful PA policy are addressed in 44% (*n* = 7) of the instruments. Items about the timeframe for policy implementation and consultations in the policy development process are included in 38% (*n* = 6) of the instruments. Evidence-based PA policy is addressed in 31% (*n* = 5) of the instruments.

Most publications excluded based on the title/abstract were (1) not related to PA/SB (e.g. publications focused on climate change, war, history, racial differences, sedentarism/nomadism, tobacco/smoking, HIV/AIDS, food, etc.), (2) epidemiological studies related to various health issues and (3) PA/SB studies that were not about policies. Most publications excluded based on their full text were (1) focused on PA/SB policies but did not describe and/or use any instrument for policy analysis, (2) described and/or used an instrument for policy analysis that focused on international, subnational, local or institutional PA/SB policies, or (3) described and/or used an instrument for the analysis of health, sport, obesity, NCD or chronic disease-related policies only, without specific reference to PA/SB policies.

### Purpose of analysis

The majority of instruments (56%; *n* = 9) were developed for both policy auditing and assessment purposes [33, 37, 38, 41–45, 48]. Five instruments (31%) were designed only for auditing purposes [34, 46, 50, 57, 58] and 2 (12%) only for assessment purposes [47, 55]. In total, 88% (*n* = 14) of the instruments contain items for auditing and 70% (*n* = 11) contain items for assessment.

### Policy sectors

Only 38% (*n* = 6) of the included instruments ask about specific sectors [33, 34, 41, 42, 55, 57]. The number of sectors addressed by these 6 instruments ranges between 1 and 11. One instrument (6%) [33] asks about policies in all 11 sectors included in the CAPPA framework. The ‘education’, ‘health’, ‘transport’ and ‘urban/rural planning and design’ sectors are the most represented sectors. These are included in 5 instruments that ask about policy sectors [33, 34, 41, 42, 57]. The ‘sport’ [33, 34, 41, 57], ‘leisure and recreation’ [33, 34, 41, 57], ‘work and employment’ [33, 41, 42, 57], and ‘public finance’ [33, 42, 55, 57] sectors are included in 25% (*n* = 4) of the instruments. The ‘environment’ sector is addressed in 19% (*n* = 3) of the instruments [33, 34, 57], the ‘research’ sector in 13% (*n* = 2) of the instruments [33, 42] and ‘tourism’ is the least represented policy sector, included in only 1 instrument (6%) [33].

### Type of policy

The reviewed instruments include items on between 1 and 3 (out of 5) different types of policy (mode = 2). Items about ‘formal written policies’ are included in 88% (*n* = 14) of the instruments [33, 34, 37, 38, 41–46, 48, 50, 57, 58], followed by items on ‘formal procedures’ in 63% (*n* = 10) [33, 34, 38, 41, 43, 45, 46, 48, 57, 58] and ‘written standards and guidelines’ in 38% (*n* = 6) [33, 34, 43–45, 57] of the instruments. None of the instruments include items on ‘unwritten formal statements’ or ‘informal policies’. Finally, 13% (*n* = 2) of the instruments do not address any specific type of policy [47, 55]; they refer to PA policy in general, without specifying the type of PA policy.

### Stages of the policy cycle

The reviewed instruments include questions on 1–4 different stages of the policy cycle (modes = 3 and 4) out of 8 possible stages included in the CAPPA framework. The majority of instruments (81%, *n* = 13) include items about the policy ‘implementation’ stage [33, 37, 38, 41, 42, 44–48, 50, 55, 57]. In total, 69% (*n* = 11) of the instruments include items about the ‘formulation’ [33, 37, 38, 41, 42, 45–48, 50, 55] and ‘evaluation’ [33, 37, 38, 41, 42, 44–46, 48, 50, 55] stages. The ‘maintenance’ stage is addressed in 3 (19%) instruments [33, 34, 50] and ‘agenda-setting’ in 2 (13%) instruments [41, 55]. Only 1 (6%) instrument includes items on the ‘endorsement/legitimisation’ stage [46]. None of the instruments include items about the ‘termination’ and ‘succession’ stages of the policy cycle. Two (13%) instruments do not include items on any particular stage of the policy cycle [43, 58].

### Scope of analysis

The instruments include items on 1–6 elements that fall within the scope of analysis according to the CAPPA framework (mode = 3). The majority of instruments (88%, *n* = 14) include items about ‘actors’ in the policy process [33, 34, 37, 38, 41–46, 48, 50, 55, 57]. Policy ‘content’ is addressed in 81% (*n* = 13) of instruments [33, 34, 37, 38, 41–46, 48, 50, 57] and policy ‘context’ in 75% (*n* = 12) [33, 34, 38, 41–45, 47, 48, 55, 57] of the instruments. Items about policy ‘processes’ [33, 37, 38, 41, 44, 45, 48] and items about ‘political will’ [33, 41, 44, 45, 47, 49, 55] are included in 44% (*n* = 7). Items about ‘availability’ of PA policies are included in 38% (*n* = 6) [33, 34, 41, 43, 57, 58] of the instruments. Items about ‘effects’ of PA policies are the least represented, as only 3 (19%) instruments include them [43, 47, 48].

## Discussion

This is the first systematic review of instruments for the analysis of national PA and SB policies. Although a relatively large number of instruments was identified, none of them cover all elements needed for a comprehensive analysis of PA/SB policy according to the CAPPA framework. Moreover, data on some important aspects of PA/SB policy, including ‘unwritten formal statements’, ‘informal policies’, the ‘termination’ stage and the ‘succession’ stage cannot be collected by any of the instruments.

All the instruments identified in the current review included items about PA policy, whilst only two asked about SB policy [33, 34]. Research on SB is a much younger field than PA research. Interest in SB as a health risk factor has been developing since 2000 [36]. While the body of evidence on determinants, prevalence, trends and health outcomes of SB is large and rapidly growing, the research on SB policies is still in its infancy [25]. Given the wide recognition of the importance of SB as a health risk factor, this area requires further development of instruments or modification of existing ones to allow for the analysis of SB policies.

The included instruments contain items for auditing or assessment of policy. Policy auditing may be considered a prerequisite for policy assessment as it is important to find out which aspects of policy exist before they can be assessed [35]. Two included instruments contain only items for PA policy assessment, implying that, if they were to be used, policy auditing first needs to be done using some other instrument [47, 55]. In order to thoroughly understand PA/SB policies, it would be beneficial if they were first audited and then assessed. Therefore, having matching items for both these purposes in a single instrument would allow for an easier and more straightforward analysis and interpretation of results. This potentially useful feature has not been found in any of the included measurement tools.

A comprehensive approach that integrates policies across settings and sectors is considered essential to achieve substantial increases in PA at the population level [9]. Cross-sectoral approaches to policy-making may assist in positioning PA promotion on the agendas of different policy levels and policy sectors [60]. In the PA policy audit of seven European countries, performed using the Health-Enhancing Physical Activity Policy Audit Tool (HEPA PAT), one of the conclusions was that supportive PA-related policies were evident in the health, education and sport sectors, but that more opportunities should be created for supportive policies in other sectors [52]. Most included instruments in this review do not ask about specific sectors. Interestingly, tourism is the least represented sector, addressed in only one instrument [33]; although some authors suggest that this sector may have great potential to contribute to PA promotion [61], this has clearly not yet been sufficiently recognised in instruments for PA policy research.

Formal written policies are, by far, the most represented type of policy in the available instruments. Accordingly, a systematic review found that formal written policies were the most commonly analysed type of national PA/SB policy globally [25]. Items about written standards and guidelines and formal procedures are also well represented in the instruments. By contrast, in the available instruments, no attention has been given to unwritten formal statements and informal policies. Including unwritten formal statements in the analysis of national PA/SB policy could bring additional insights into the comprehensive decision-making processes. As already recognised by Schmid et al. [62] informal policies are “*considered to be part of culture rather than explicit policy and not a primary focus of initial physical activity policy research*”. Rütten at al [49]. based their instrument on a broader definition of policy, stating that, besides formal statements and procedures, policy also includes informal procedures, rationales for action and arrangements. However, this was not explicitly reflected in the instrument’s items.

In political science, usually, at least five stages are mentioned as crucial for understanding the full life circle of a policy and making sense of the policy process as a whole [63, 64]. Within most reviewed instruments, only a partial, three-stage policy cycle is inquired about, including the development of policy (formulation stage), policy implementation and the evaluation stage. We found only one instrument that includes an item on the endorsement/legitimisation stage of PA policies, which is not surprising given there does not seem to have been much interest in this particular aspect of policy in previous research in this field [25]. It is also possible, however, that the selection of research topics has been determined by the availability of measures. The agenda-setting and maintenance stages are addressed in only a few instruments, while none of the instruments address the termination and succession stages. Analysing PA/SB policy in the context of its full policy cycle, from agenda-setting to the termination or succession stage, is important to gain a more thorough understanding of the whole PA/SB policy-making process.

The majority of instruments are focused on policy content and the actors involved in policy processes. Some of the most common items on actors across the instruments are focused on leadership, coordination mechanisms and organisational structure for PA promotion. Some of the most common items related to policy content are about the target groups and policy’s specific goals and objectives. Only a few instruments ask about the availability of PA/SB policies, that is, analysis of whether a specific PA/SB policy exists or not [35]. With regards to the analysis of processes related to PA/SB policy, instruments that include relevant items are mainly focused on the processes of collaboration and/or consultation regarding PA policy. However, a detailed analysis of processes can be performed with very few instruments. For example, little attention has been given to actions and interrelationships between various actors and to formal processes during the development and implementation of policy. Besides, none of the instruments ask about the power relationship in different processes.

The context surrounding policy is addressed in most of the instruments and the respective items focus on the budget/financial resources and political will/support regarding policy implementation. Assessing the national policy context is a significant first step to better PA policy [52]. However, broader, country-specific context, such as religious, social or other values relevant for PA promotion, dominant ideology, and the nature of political systems, was addressed by very few instruments. An examination of a narrow context specifically focused on economic and political circumstances relevant for PA policy may be misleading. If, for example, a researcher does not consider the dominant values of a country, they may be missing the ‘full picture’ relevant to understanding how PA promotion in that country really works.

Analysing political, public health, social, economic and/or environmental impacts is one of the key aspects of policy analysis. However, we found only a few instruments that include items about the effects of PA policy. This aspect of PA policy analysis may have been neglected because the effects of PA policies can be complex and challenging to measure. In 2006, the Centers for Disease Control and Prevention highlighted that their “*first priority*” for future research was “*to develop better tools to assess the effects of policies*” [62]. Milton and Bauman [65] also noted that evaluating the effectiveness of PA policy is important to inform future policy development. Such endeavours could be supported by the development of instruments specialised for analysing the effects of PA and SB policies.

### Recommendations for the use of instruments for PA/SB policy analysis

We suggest to future users of the instruments, such as policy analysts, policy-makers and other stakeholders, to first use the CAPPA framework as a ‘road map’ to determine a more specific ‘route’ to answer their research question [35]. This can help to inform decisions on which particular instrument best meets their needs. All instruments assessed in this review have advantages and disadvantages.

If a comprehensive PA policy analysis needs to be done, HEPA PAT would be the most suitable instrument. Using such a comprehensive instrument has advantages in that it can (1) provide a deeper understanding of the current state of national PA/SB policies and (2) lead to a more detailed insight on what needs to be changed in order to improve policy development and/or implementation. On the other hand, using a comprehensive instrument usually means longer data collection, which may slow down the process of policy analysis, and once the analysis is finally completed, it may already be outdated. According to some experts who are currently using HEPA PAT, if undertaken by a single researcher, the process can take up to more than a year. Therefore, we believe this instrument is especially suitable for an official governmental audit of national PA/SB policy where a team of people is available to work on collecting and analysing the data.

While the HEPA PAT does have one assessment-type question, it is more suitable for an audit than for assessment. Therefore, for assessment purposes, we recommend using the Analysis of Determinants of Policy Impact (ADEPT) Model [48, 49]. This instrument is especially suitable for researchers who wish to conduct interviews with policy-makers. However, the instrument does not mention SB policies, and it relies on a broad definition of policy, which may not be suitable for some researchers who want to use a narrower definition.

It may not always be practical to conduct a comprehensive analysis of PA policy. In such cases, a less comprehensive instrument may need to be considered, albeit on account of gathering less detailed information about a PA/SB policy. If time or capacity is limited, we recommend using the GoPA! Policy Inventory [34]. It contains only 10 questions and is based on HEPA PAT – version 2 [33] and the Questionnaire on the monitoring framework for the implementation of policies to promote health-enhancing physical activity in the EU and WHO European Region 2015 [56].

Some of the instruments are not structured as questionnaires. An example are the eight policy principles for the promotion of healthy diets and PA developed by WHO [37]. If needed for the purpose of data collection, such sets of principles can be easily transformed into questionnaire items. We provided sample questions derived from the WHO’s set of principles in Additional file 3. It should be noted, however, that these sample questions have not been developed by the authors of the original instrument and their measurement properties have not been assessed. Rather, these sample questions have been developed exclusively for the purpose of this review to help readers understand how a set of criteria can be transformed into a format suitable for data collection. Depending on their study design, researchers may prefer to develop different questions and use different types of response scales. In any case, it would be important to conduct a study of measurement properties of such newly developed questions before starting the data collection.

All these recommendations are an informed opinion of the authors of this review and should not be taken as an exclusive suggestion to use one instrument over another. The final decision should be left to users, who should independently asses all instruments and decide which is the most suitable for their needs. Table 1 and Additional file 2 can help to facilitate this process.

### Towards standardisation of PA/SB policy analysis

The reviewed instruments differ considerably in their structure and comprehensiveness as well as on the aspects of policy they inquire about. This is not surprising, as there is still no consensus among political scientists on what is defined as ‘policy’ and what constitutes a good policy analysis. Somewhat surprising, however, is the fact that there were large discrepancies even between the instruments developed by the same organisation and/or the same group of authors. This clearly shows that further efforts are needed towards standardisation of PA/SB policy analysis. Despite the large differences between instruments, some themes, such as funding, specific target groups, political leadership and coordination, multi-sectoral approaches, evaluation, surveillance/monitoring, setting specific goals for PA promotion, and involvement of various stakeholders in PA policy, were found in most of them. This is promising as it suggests a certain level of agreement between researchers about items that are critical for conducting a PA/SB policy analysis. However, there are several reasons for conducting policy analysis and different instruments have been developed for different purposes. Differences between questionnaire items and conceptualisations of PA/SB policy can negatively affect the comparability of findings across studies. Nonetheless, diversity in methodological approaches may sometimes be considered desirable, particularly in younger fields like SB policy research, because it may serve as a catalyst for academic discussions and facilitate the search for optimal solutions, whereas advancing to standardisation too soon might hinder the development of some novel and potentially valuable methods. Therefore, a balanced approach between heading towards standardisation and allowing for diversity in methodological approaches may be a good way to progress PA/SB policy research.

### Strengths and limitations of the review

The main strengths of this systematic review are that (1) the search was performed through various bibliographic databases, search engines and websites as well as through the reference lists of all included publications, which reduced the possibility of missing relevant studies; (2) we employed an inclusive search syntax and broad eligibility criteria, which allowed us to find and review various types of instruments that may be used for PA/SB policy analysis; (3) the assessment of eligibility of studies as well as the data extraction from the studies were done in duplicate, which reduced the likelihood of human error and subjectivity; and (4) we based our data extraction on a conceptual framework.

This systematic review is also subject to several limitations. Even though the search was done with no language restrictions, we included only publications with abstracts and/or full-texts in English, which may have led to the exclusion of relevant studies. We focused only on national-level policies, yet we acknowledge that some instruments included in this review may also be used to analyse policies on other levels. We did not conduct a formal quality assessment of the studies and/or instruments given that the included studies varied in their aims and methods. Nevertheless, we provided a general assessment of the instruments and the strengths and limitations of various approaches employed in these.

## Conclusions

There is a range of different instruments available that can be used for analysing PA policy, whilst only two instruments include questions about SB policy. None of the instruments allow for the analysis of all the relevant components of a national PA/SB policy. Some important elements of PA policy analysis, such as the tourism and research sectors, the agenda-setting and endorsement/legitimisation stages, and the effects of policy, are addressed by only a few instruments. Moreover, none of the instruments address unwritten formal statements, informal policies, and the termination and succession stages of the policy cycle. Thus, designing new instruments or adapting existing ones is needed to allow for a more thorough analysis of national PA and SB policies. Given that policy analysis covering all important components of PA/SB policy may be extremely time-consuming, a way forward might be to develop a set of complementary instruments, with each tool collecting detailed information about a specific aspect of PA and SB policy.

## Supplementary information

**Additional file 1.** Full search syntax used for each database.

**Additional file 2.** Instruments for the analysis of physical activity and/or sedentary behaviour policies and their description.

**Additional file 3.** Sample questions for physical activity policy auditing/assessment.

## Data Availability

Full search syntax used for each database is available in Additional file 1. Full descriptions of the instruments and included publications are available in Additional file 2. Sample questions for physical activity policy auditing/assessment derived from one set of criteria are available in Additional file 3.

## Abbreviations

CAPPA: Comprehensive Analysis of Policy on Physical Activity
EU: European Union
GoPA!: Global Observatory for Physical Activity
HEPA PAT: Health-Enhancing Physical Activity Policy Audit Tool
NCD: non-communicable disease
PA: physical activity
SB: sedentary behaviour
