# Supplementary material for: A systematic review of instruments for the analysis of national-level physical activity and sedentary behaviour policies
Source: Health Res Policy Syst. 2019 Nov 13;17:86. doi: 10.1186/s12961-019-0492-4 (PMC6854623; doi:10.1186/s12961-019-0492-4)
Supplement: Supplementary file 2 — Additional file 2. Instruments for the analysis of physical activity and/or sedentary behaviour policies and their description. [file 12961_2019_492_MOESM2_ESM.docx]

**Instruments for the analysis of physical activity and/or sedentary behaviour policies and their description**

| **Instrument** | **Author(s) and**  **publication** | **Short description of the publication(s) and the instrument** | **Detailed description and/or visual representation of the instrument** |
| --- | --- | --- | --- |
| *Policy principles for the promotion of healthy diets and physical activity* | - World Health Organization, 2003 [52] | This publication is an extensive technical report of the WHO and the United Nation’s Food and Agriculture Organization expert consultation that took place in 2002 in Geneva, Switzerland. The consultation was about diet, nutrition, and the prevention of chronic diseases. The report mentions policy principles for the promotion of healthy diets and PA. | “Policy principles for the promotion of healthy diets and physical activity:  1) Strategies should be *comprehensive* and address all major dietary and physical activity risks for chronic diseases together, alongside other risks - such as tobacco use - from a multisectoral perspective.  2) Each country should select what will constitute the *optimal mix of actions* that are in accord with national capabilities, laws and economic realities.  3) *Governments have a central steering role* in developing strategies, ensuring that actions are implemented and monitoring their impact over the long term.  4) *Ministries of health have a crucial convening role* - bringing together other ministries needed for effective policy design and implementation.  5) *Governments need to work together with* the private sector, health professional bodies, consumer groups, academics, the research community and other nongovernmental bodies if sustained progress is to occur.  6) *A life-course perspective* on chronic disease prevention and control is critical. This starts with maternal and child health, nutrition and care practices, and carries through to school and workplace environments, access to preventive health and primary care, as well as community based care for the elderly and disabled people.  7) Strategies should explicitly address equality and diminish disparities; they should focus on the needs of the *poorest communities and population groups* - this requires a strong role for government. Furthermore, since women generally make decisions about household  nutrition, strategies should be *gender* sensitive.  8) Strategies need to draw substantially on existing *international standards* that provide a reference in international trade… WHO’s international leadership role in pushing forward the agenda on diet, physical activity and health is crucial.” p. 135-136 |
| *Criteria for successful policy and action plans on physical activity* | - Bull et al., 2004 [47]  - Bull et al., 2004 [58]  - Schöppe et al., 2004 [56] | This study contains a journal article and two reports. It is an analysis of the development and the content of PA policy several countries around the world. The data on policies were extracted from articles, national policy documents, and grey literature obtained through an electronic literature search. PA policies of several countries around the world were assessed against 11 specific criteria for successful PA policy and action plans. | “Criteria for successful policy and action plans on physical activity:   1. *Consultation with* key stakeholders during development of policy and action plans; 2. Adoption of a comprehensive approach using *multiple strategies* (eg, individual-oriented as well as environmental focused interventions) targeting different population groups; 3. Working at *different levels* (local, state and national as well as individual whole community and physical environmental level); 4. Development of policy and action plan across multiple agencies by *working through coalitions, alliances and partnerships* (eg, involving cross government, non-government as well as relevant private sector partners); 5. *Integration* of PA policy within other related agendas (eg, in the field of health, nutrition, transport, environment); 6. *Stable base of support and sustainable resources* to implement the policy and action plan; 7. Development of an *Identity* for the policy and action plan by means of a logo, branding and/or slogan and/or key spokesperson or 'champion' for the initiatives as well as an advocacy / communication plan; 8. A clear statement of the *Timeframe* of the policy commitment and implementation of the action plan; 9. Specific plans and resources for *Evaluation* of the policy and action plan implementation (undertaking evidence based approaches supported by appropriate budget);   10) Development and/or maintenance of appropriate *Surveillance or Health Monitoring Systems* including measures of levels of physical (in)activity;  11) Statement of recognition of existing *National guidelines/recommendations on PA* or intent to develop them.” [47] p. 96 |
| *A Comprehensive Physical Activity Policy Framework* | - Shephard et al., 2004 [41] | This is a practice article on WHO/CDC consultation on PA policy development that took place in Atlanta, Georgia in 2002. The article outlines the context and outcomes of the consultation. It describes a comprehensive, six- stage PA policy framework. | 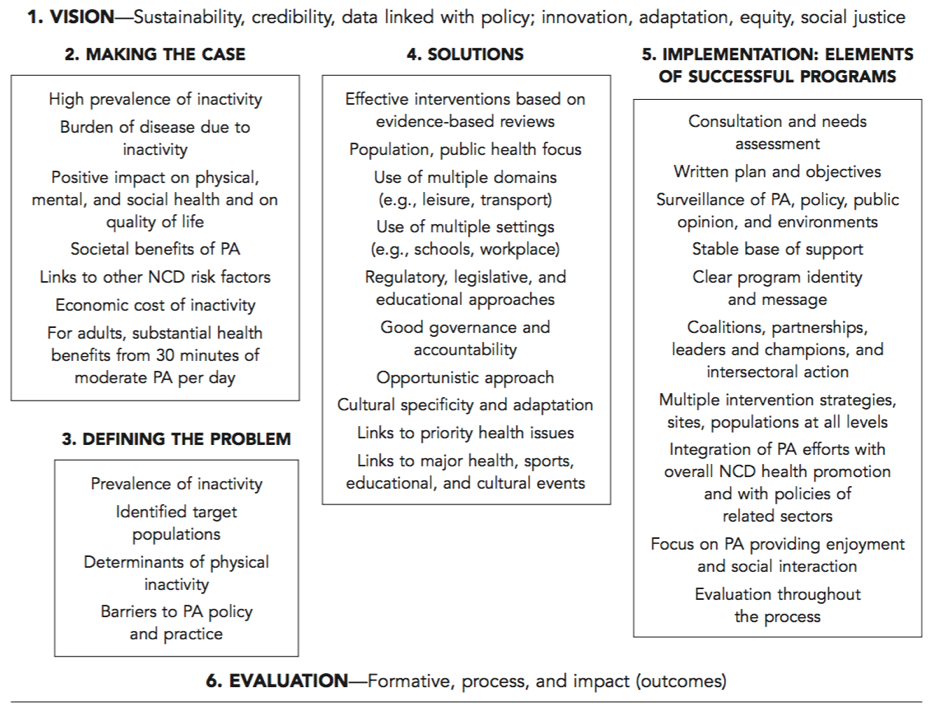  A Comprehensive Physical Activity Policy Framework p. 348 |
| *Elements of national policy documents* | - Branca et al., 2007 [49] | This publication issued by the WHO Regional Office for Europe is about the obesity challenge in the WHO European Region. One of its chapters is related to *National policies in the European Region*. National policy documents on PA, obesity, and nutrition were analysed using an analytical framework based on elements used in previous policy analyses and policy analysis tools. | *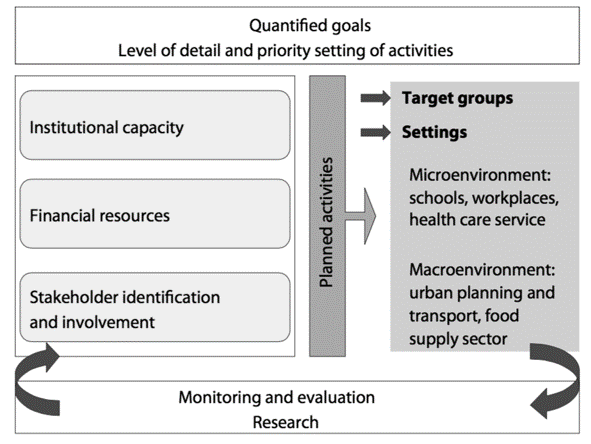*  Elements of national policy documents p. 260 |
| *Key principles that should guide member states in the development of national physical activity strategies* | - World Health Organization, 2007 [53] | This publication issued by the WHO Regional Office for Europe provides a guidance for the WHO member states, policy-makers, and experts on designing and implementing PA-promoting policy and action. It provides seven key principles for successful PA strategies applicable at national and subnational levels. | 1) Population health: The population health approach (PHA) focuses on improving the overall health status of the population and subpopulations, rather than on the individual. While the ultimate goal is to improve the health of individuals, the PHA emphasizes improving the broad conditions and environments that affect health and healthy choices. This includes addressing some of the root causes that lead to poor health outcomes, reducing inequities in health status between subpopulations, increasing awareness of healthy choices, and creating services and environments that promote and maintain health.  2) Comprehensive: Strategies should include components such as public awareness, multiple physical activity interventions and patterns, leadership development, active infrastructure development and renewal, policies development, and partnership building. Strategies should consider initiatives in settings including the home/family, community, school, workplace and health care system. Strategies should focus on target groups, with an emphasis on the inactive, while not forgetting the general population and those already active.  3) Integrated: Many strategies are undertaken in isolation and do not benefit from the value and efficiencies of integrated approaches. Integration should be both vertical and horizontal. Vertical integration includes a seamless flow of information and involvement at the national, regional and local levels. Horizontal integration between areas such as health, education, transport, urban planning, recreation, social services and sport is just as vital. National and regional strategies need to support efforts at the local level where the greatest capacity for impact exists.  4) Complementary and collaborative: Physical activity is a leading lifestyle health determinant, and is a vital component in addressing obesity. Physical activity strategies should be linked to those focused on healthy eating and other health and chronic disease risk factors; communication campaign messages should be complementary where appropriate. Strategies at all levels require collaborative approaches by governmental, voluntary and corporate sectors. Those responsible for planning and implementing strategies should represent various sectors and areas, and seek out opportunities to act collaboratively.  5) Sustainable: Political and organizational commitment to long-term physical activity strategies is required at all levels. Local strategies should be supported by national strategies, but should not be financially dependent on them. The most effective national supports include leadership development, resources and tools, research and evaluation, proportional contributions toward active infrastructure development, public policies that enable local action and collaboration, and communication campaigns that can be complemented by local efforts. Sustainability is rooted in local commitments to ongoing actions and investment in planning, programmes, facilities, open spaces and active transportation systems.  6) Evidence-based and effective: National strategies need to identify clear outcomes that are based on measurable change. Their outcomes may include increasing the physical activity levels of the population and reducing obesity levels. Realistic targets should be set for these outcomes over specific time frames. Mechanisms need to be in place to monitor ongoing progress and to provide timely and meaningful data on the results. The national strategy should integrate research and evaluation into its program and policy development, and help communities to carry out meaningful measurement and evaluation on their own. The results of evidence-based interventions and other related research should be synthesized and disseminated on an ongoing basis.  7) Communicated: National physical activity strategies should consider the development of high-profile communication campaigns that increase overall awareness of the benefits of physical activity and encourage behaviour change. The messages should be consistent and clear, with many targeted at key population segments. National communication strategies should support local campaigns and be flexible enough to be used on multiple channels. They should also create networks to allow communities to share plans and success stories. The Member States also need to develop mechanisms for ongoing communication and information sharing regarding their strategies.  p. 17-18  The publication also states recommendations for focused national commitment that should ensure that capacity is built up in terms of: “human resources with adequate skills and competencies; an organizational structure reflecting the importance of this health determinant; appropriate regulations, including legislation; national guidelines; a national action plan for physical activity; national programmes and campaigns; an intersectoral approach achieved through cooperation between different ministries; economic resources.” p. 20 |
| *Important elements of successful physical activity policies and plans* | - World Health Organization, 2007 [51] | This publication issued by the WHO is a guide that aims to provide guidance for an effective PA promotion and assist in the implementation and development of national PA plans. The guide was initially developed by the participants of the WHO workshop in 2005 in China. It contains 18 important elements of successful policies and plans for the WHO member states to into account when developing and/or implementing their PA policies. | “Important elements of successful policies and plans:   1. High-level political commitment; 2. Integration in national policies; 3. Identification of national goals and objectives; 4. Overall health goals; 5. Objectives; 6. Funding; 7. Support from stakeholders; 8. Cultural sensitivity; 9. Integration of physical activity within other related sectors;   10) A coordinating team;  11) Multiple intervention strategies;  12) Target whole population as well as specific population groups;  13) Clear identity;  14) Implementation at different levels within “local reality”;  15) Leadership and workforce development;  16) Dissemination;  17) Monitoring and evaluation;  18) National physical activity guidelines.” p. 3-8 |
| *HARDWIRED criteria for successful national physical activity policy* | - Bellew et al., 2008 [46] | This journal article provides a comparison of Australian PA policy with international PA policies in seven countries assessed using nine criteria for successful national physical activity policy, the so called HARDWIRED criteria. Literature and policy reviews were combined with questionnaires sent to purposively sampled experts. | “Criteria for successful national physical activity policy (HARDWIRED):   1. Highly consultative in development; 2. Active through multi-strategic, multi-level, partnerships; 3. Resourced adequately; 4. Developed in stand-alone and synergistic policy modes; 5. Widely communicated; 6. Independently evaluated; 7. Role-clarified and performance-delineated; 8. Evidence-informed and Evidence-generating; and 9. Defined national guidelines for health enhancing physical activity.” p. 2 |
| *Eight aspects identified as being relevant for effective physical activity policies* | - Daugbjerg et al., 2009 [40] | This journal article is a content analysis of 27 policy documents from 14 countries in the European region. The documents were analysed based on eight aspects being relevant for effective PA policies. | Aspects “identified as being relevant for effective physical activity policies:   1. Sectors and institutions involved: involvement of different sectors in the preparation and implementation of the policy. Important partners include various sectors of the national government, subnational authorities, municipalities, nongovernmental organizations, the private sector, the media, associations, educational institutions, employers, etc.; 2. Implementation: implementation plan for the policy and a clear definition of the body or bodies responsible for the implementation; 3. Legal status: legally binding or nonbinding; formally adopted by government or not; 4. Target groups: clearly identified population groups targeted by the policy; 5. Goals and targets: physical activity goals or targets were specified for certain population groups and time periods; 6. Timeframe: clear timeframe specified for the implementation of the policy; 7. Budget: specified budget allocated to the implement of the policy; 8. Evaluation and surveillance: development or continuation of an evaluation on the implementation and results of the policy; surveillance or monitoring system to measure physical activity.” p. 807 |
| *A graphical, computer-*  *based decision-*  *support tool to help*  *decision makers*  *evaluate policy*  *options relating to*  *physical activity* | - Yancey et al., 2010 [45] | This study described the development of a computer-based Decision-Support Tool. It was developed as a response to a request from the California Department of Health Services to provide assistance on assessment of policies for PA promotion. The tool is designed to support policy makers to systematically weigh, compare, and synthesise evidence specific to public-health relevant policies and interventions. | Decision Support tools for public health policies and interventions and its criteria:  “Feasibility:  1) Low start-up costs  2) Quickly implementable  3) Political will/community receptivity  4) Reliability, consistency  5) Likelihood of sustainability  6) Availability of critical adjuncts to realize effects  7) User-specified Feasibility criteria  Evidence:  8) Quality and quantity of scientific evidence  Population impact:  9) Short-term efficacy  10) Effectiveness in general population  11) Effectiveness in target population (specificity)  12) Effectiveness in saving aggregate Quality-Adjusted Life-Years (QALYs)  13) Secondary health benefits (e.g. improved nutrition from increased physical activity)  14) Potential Dose Effects  15) Cost-effectiveness  16) Potential cumulative effects as part of a larger, coordinated strategy  17) User-specified Impact criteria  Disparities reduction:  18) Magnitude of aggregate health effects (benefits) in high-risk or target populations  19) Reductions in existing disparities due to differential utilization or uptake  20) Proximal and distal distributional effects on different population segments  21) User-specified disparities reduction criteria” p. 275 |
| *Analysis of Determinants of Policy Impact (ADEPT) Model* | - Rütten et al., 2010 [44]  - Rütten et al., 2012 [43] | In Rütten et al., 2010 The Analysis of Determinants of Policy Impact (ADEPT) approach is presented. The ADEPT model aims to explain and influence policy impact implementation and policy development using four determinants: obligations, goals, resources, and opportunities. It is a theory-based tool for influencing and understanding policy processes in health promotion. An empirical testing of the ADEPT model was conducted using a quantitative survey of policy makers from six European countries and from four health promotion policy fields.  Rütten et al., 2012 study tested a theoretical model to develop and assess policies for PA promotion among older adults. For this study, a short 14-item List for Policy Assessment in Health Promotion version of the original questionnaire, was used for PA policy assessment. The questionnaire assessed policy-maker’s perceptions of the organisational resources, goals, opportunities and obligations in the area of PA promotion. | “List of items of items operationalizing ADEPT:  Policy determinants  Goals  The goals are officially spelled out  The goals are concrete enough  The action centers on improving the health of the population  Obligations  Personally I feel obliged to do something in this field  The action is part of my professional duties  Scientific results demand the action  We are obliged to the population to act in this area  Resources  There is enough personnel  My organization has the necessary capacities  There are sufficient financial resources  Organizational opportunities  My own involvement has worsened/improved  The co-operation within my organization has worsened/improved  Political opportunities  The political climate has worsened/improved  The support from other sectors has worsened/ improved  The co-operation between political levels involved has worsened/improved  The co-operation between public and private organizations has worsened/improved  The lobby for the action has worsened/improved  Public opportunities  The involvement of the population has worsened/ improved  The population supports the action  The media’s interest has worsened/improved  Policy impact  Outcome  The action has achieved the intended behavior change in the population  Considering cost-benefits, the action was worthwhile  Personally I am satisfied with the results  Output  Various programs were implemented” [44] p. 325 |
| *Categories for the content analysis of policies* | - World Health Organization, 2011 [50]  - Christiansen et al., 2014 [39] | The study contains one journal article and one WHO publication. It used policy content analysis of EU member states’ national (and subnational if no national available) policy documents related to HEPA and sport. The content analysis grid was developed based on Daugbjerg et al. (2009) and Bellew et al (2008). The grid was complemented with indicators relating specifically to sport. | “The following categories used for the content analysis of the policies:   1. General information – information about country of origin, language, issuing body and publication year 2. Timeframe – was a clear timeframe specified for the implementation of the document; 3. Stakeholder involvement in the development phase – the process of involving different stakeholders in the development of the strategies; 4. Reference to other national/international documents or physical activity guidelines – whether reference was made to other national or international documents; 5. Sport participation and health-enhancing physical activity – whether goals and targets were set for increasing sport participation and/or health-enhancing physical activity levels; 6. Elite sport and sport for all – whether elite sport and/or sport for all were addressed in the strategy; 7. Infrastructure – whether sport infrastructure was addressed in the strategy; 8. Target groups – which population groups were targeted by the strategy; 9. Settings – which settings are addressed (e.g. schools, workplaces); 10. Implementation – the body responsible for implementation, whether other roles and responsibilities were outlined, and whether local-level implementation was addressed   11) Budget – whether a specified budget was allocated to implement the policy; and  12) Evaluation – whether the strategy had an evaluation plan and whether the main responsibility for evaluation was clarified.” [39] p. 53-54 |
| *Health-enhancing physical activity (HEPA) policy audit tool (PAT)* | - Bull et al., 2014 [17]  - Bull et al., 2014 [42]  - Bull et al., 2014 [54]  - Bull et al., 2014 [55]  - Bull et al., 2015 [33] | The study contains two journal articles and two technical reports. It compared the development processes, content, and implementation of HEPA policies in seven EU countries (Finland, Italy, the Netherlands, Norway, Portugal, Slovenia, Switzerland). The data was collected using a 27-item instrument for PA policy audit - Health Enhancing Physical Activity Policy Audit Tool (HEPA PAT). For each country, a leading academic, a representative of relevant institute or (sub)national government official, was in charge for completion of the HEPA PAT. Directed content analysis was used to analyse collected data. A full description of the development of the HEPA PAT was described in one of the journal articles. The HEPA PAT is structured around 17 criteria identified as successful elements for policy approaches to PA. | Eleven sections of the HEPA PAT:  “1) Background information and country context, including government structure  (e.g. Please provide a brief overview of the government structure in your country)  2) Leadership and partnerships for HEPA promotion  (e.g. Please state any agency(ies) providing leadership for HEPA promotion at the national level in your country.)  3) Key policy documents and their development process, including country history of physical activity policy  (e.g. Please describe any key past policy documents and past events that have led to the current context of HEPA promotion in your country)  4) Scope and content of relevant policies and examples of implementation  (e.g. Considering all the key physical activity policy documents listed in Question 7, please indicate which settings are included for the delivery of specific HEPA actions.)  5) Recommendations, goals and targets  (e.g. Does your country have any national recommendations on physical activity and health?)  6) Surveillance  (e.g. Does your country have a health surveillance or monitoring system that includes measures of physical activity or sedentary behaviour?)  7) Evaluation of relevant policies  (e.g. Has your country undertaken evaluation of any of the national policies or action plans listed in Question 7?)  8) Funding and political commitment  (e.g. Within each of the sectors listed, is funding specifically allocated or “ring-fenced” for the delivery of physical activity- related policy or action plans at the national level?)  9) Capacity building through a national network  (e.g. Does any professional network or system exist in your country that links and/or supports professionals interested or currently working in physical activity or related areas?)  10) Experience of policy implementation, progress and remaining challenges  (e.g. What do you think are the areas of greatest progress in national HEPA promotion in your country in recent years?)  11) Summary of the process undertaken to complete an assessment using the HEPA PAT” [33] p. 2 |
| *Government Strategies and Investments* *indicator for Active Healthy Kids Report cards* | - Tremblay et al., 2014 [38] | This journal article contains consolidated findings of Report Cards (RC) on PA in children and youth. The RCs include an assessment of wide range of PA indicators for children and youth. Each indicator was assessed according to its respective benchmarks by the group of experts in charge of their country’s RC. | Benchmarks used to guide the *Government Strategies and Investments* indicator:  “1) Evidence of leadership and commitment in providing physical activity opportunities for all children and youth.  2) Allocated funds and resources for the implementation of physical activity promotion strategies and initiatives for all children and youth.  3) Demonstrated progress through the key stages of public policy making, that is policy agenda, policy formation, policy implementation, policy evaluation, and decisions about the future.” p. S116  Indicators and benchmark tools are available at:  <https://www.activehealthykids.org/tools/> |
| *Questionnaire on*  *the monitoring*  *framework for*  *the implementation*  *of policies to*  *promote health-enhancing physical activity in the EU and WHO European Region* 2015 | - World Health Organization, 2015 [48]  - European Physical Activity Focal Points Network, 2015 [59] | This publication issued by the WHO Regional Office for Europe contains country factsheets related to HEPA promotion in the EU. It is an overview of information related to monitoring, surveillance, and policy response. The WHO/EU PA focal points provided and validated PA-related data for their countries. For each member state a “country profile” was created from the data collected using a questionnaire on 23 HEPA indicators. | Indicators of the *Questionnaire on the monitoring framework for the implementation of policies to*  *promote health-enhancing physical activity in the EU and WHO European Region*:  “Indicator 1: National recommendation on physical activity for health  (e.g. *Does a national recommendation on physical activity and health exist in your*  *country, i.e. an officially adopted statement on the duration, intensity and frequency of*  *physical activity behavior that the population should reach?)*  Indicator 4: National government coordination mechanism and leadership on HEPA promotion  (e.g. *Has a specific coordinating mechanism (e.g. working group, advisory board, coordinating institution etc.) been developed for HEPA promotion in your country?*  Indicator 5: Funding allocated specifically to HEPA promotion  *(e.g. What is the yearly funding (in national currency) allocated specifically to HEPA promotion?)*  Indicator 6*: National Sport for All policy or action plan  Indicator 9*: Target groups addressed by the national HEPA policy  Indicator 22*: National HEPA policies that include a plan for evaluation  *(e.g. Does your country have a national policy and/or a national action plan on Sport for All promotion?; Which target groups does/do the national HEPA promotion policy/policies address, especially regarding groups in particular need of physical activity?; What is the percentage of national HEPA policies that include a clear intention or plan for evaluation?*)  Indicator 10: Monitoring and surveillance of physical activity  *(e.g. Does your country have an established surveillance or health monitoring system that includes population-based measures of physical activity?)*  Indicator 11: Counseling on physical activity  (e.g. *Does a programme or scheme to promote counseling on physical activity by health professionals exist in your country?)*  *Indicator 12: Training on physical activity in curriculum for health professionals*  *(e.g. Is physical activity and health (health effects, determinants, effective interventions etc.) taught in a module of the curriculum of health professionals, incl. e.g. nurses, doctors, physiotherapists?)*  Indicator 13: Physical education in primary and secondary schools  *(e.g. What is the number of hours of physical education provided in primary schools?)*  Indicator 14: Schemes for school-related physical activity promotion  (e.g. *Does your country have a national scheme for active school breaks (i.e. breaks between school lessons)?)*  Indicator 16: Schemes promoting active travel to school  (e.g. *Does a national scheme exist to promote active travel to school (e.g. walking buses, cycling)?)*  Indicator 17: Level of cycling and walking  *(e.g. Does your country use tax incentives to promote active transport (such as congestion charges, increased parking fees or motor vehicle taxes)?)*  Indicator 19: Schemes to promote active travel to work  *(e.g. Does a national scheme exist to promote active travel to work (e.g. walking, cycling)?)*  Indicator 20: Schemes to promote physical activity at the workplace  (e.g. *Does a national scheme exist to promote physical activity at the work place?)*  Indicator 21: Schemes for community interventions to promote physical activity in older adults  *(e.g. Does a specific national scheme or programme for community interventions to promote physical activity in older adults exist in your country?)*  Indicator 23: National awareness raising campaign on physical activity  *(e.g. Does a clearly formulated national campaign for physical education and public awareness exist in your country?)*  * Indicators six, nine, and 22 were combined together in the questionnaire”. [59] p. 4-36 {European Commission, 2013 #57852;European Physical Activity Focal Points Network, 2015 #57901} |
| *Surveillance and Policy status indicators for GoPA Country Cards* | - Ramirez Varela et al., 2016 [57]  - Ramirez Varela et al., 2017 [37] | This almanac and the associated journal article contain Report Cards (RC) on PA for 217 countries. Each RC contains six common indicators: general information; physical activity prevalence; physical inactivity health burden and related mortality; national physical activity plan; physical activity surveillance; and research in physical activity. Data were obtained through Internet search. Data for 139 countries were reviewed and approved by country contacts. The RCs reported on the availability of national or sub-national PA plans, which was one of six PA indicators. | Surveillance and policy status:  Content of the fourth indicator *National plan*:  “Availability of a national or sub-national PA plan, classified as:  a) no clear physical activity policy  b) physical activity embedded as part of a NCD plan  c) standalone physical activity plan”  Content of the fifth indicator surveillance:  “Existence of a national survey that includes physical activity questions and the first, most recent and next survey, classified as:  a) no national physical activity surveillance data  b) one physical activity survey identified  c) two surveys identified  d) three or more surveys identified, and a clear periodicity, with a specific year for the next survey”. [57] [57] p. 28 |
| *GoPA Policy Inventory 1.0 2017* | - Global Observatory for Physical activity, 2017 [34] | This questionnaire is still unpublished document developed by the GoPA. It is based on the second version of the HEPA PAT and the European Monitoring Framework. It contains ten items. | Sections of the questionnaire:  1) Main government ministries that have active role in HEPA promotion;  2) Important national organizations (outside government) actively engaged in HEPA promotion;  3) Documents that outline intention to increase national PA levels (policy documents, legislation, strategies or action plans);  4) Existence and target groups of national recommendations on PA and health;  5) Existence and target groups of national recommendations on SB reduction;  6) Existence of surveillance/monitoring system;  7) Existence of national goals and timeframe for increasing PA levels;  8) Settings included in delivering of HEPA actions;  9) Population groups targeted by HEPA actions;  10) Existence of professional network for supporting professionals working in PA area.” |
