# Supplementary material for: A systematic review of instruments for the analysis of national-level physical activity and sedentary behaviour policies
Source: Health Res Policy Syst. 2019 Nov 13;17:86. doi: 10.1186/s12961-019-0492-4 (PMC6854623; doi:10.1186/s12961-019-0492-4)
Supplement: Supplementary file 3 — Additional file 3. Sample questions for physical activity policy auditing/assessment. [file 12961_2019_492_MOESM3_ESM.docx]

**Additional file 3 - Sample questions for PA policy assessment/auditing**

Sample questions are derived from the following set of criteria:

World Health Organization, *Diet, nutrition and the prevention of chronic diseases, Report of a Joint WHO/FAO Expert Consultation*. 2003, World Health Organization: Geneva.

| Sample questions 1 and 2 are derived from the following principle: “Strategies should be *comprehensive* and address all major dietary and physical activity risks for chronic diseases together, alongside other risks - such as tobacco use - from a multisectoral perspective”. [1] |
| --- |

1. On the scale from 1 to 5, please rate how comprehensive is the national PA strategy (or an equivalent policy document)!

🞅 1 – not at all comprehensive

🞅 2

🞅 3

🞅 4

🞅 5 – very comprehensive

🞅 Don’t know

1. Does the national PA strategy (or an equivalent policy document) address the problem of insufficient physical activity from a multisectoral perspective?

🞅 Yes

🞅 No

🞅 Don’t know

| Sample questions 3 and 4 are derived from the following principle: “Each country should select what will constitute the *optimal mix of actions* that are in accord with national capabilities, laws and economic realities”. [1] |
| --- |

1. When addressing the problem of insufficient PA, did your country select an optimal mix of actions?

🞅 Yes

🞅 No

🞅 Don’t know

1. Were the selected actions in accordance with…

| … national capabilities? | 🞅 Yes | 🞅 No | 🞅 Don’t know |
| --- | --- | --- | --- |
| … national laws? | 🞅 Yes | 🞅 No | 🞅 Don’t know |
| … national economic realities? | 🞅 Yes | 🞅 No | 🞅 Don’t know |

| Sample questions 5, 6, 7, 8, and 9 are derived from the following principles*:* “*Governments have a central steering role* in developing strategies, ensuring that actions are implemented and monitoring their impact over the long term”. [1] |
| --- |

1. Which body had a central steering role in the development of the national PA strategy (or an equivalent policy document)?

🞅 _____________________________________________________________

🞅 Don’t know

1. Is it ensured that the actions specified in the national PA strategy (or an equivalent policy document) are implemented?

🞅 Yes

🞅 No

🞅 Don’t know

1. Which body ensures that the actions specified in the national PA strategy (or an equivalent policy document) are implemented?

🞅 _____________________________________________________________

🞅 Don’t know

1. Is it ensured that the impact of the actions specified in the national PA strategy (or an equivalent policy document) is monitored over the long term?

🞅 Yes

🞅 No

🞅 Don’t know

1. Which body ensures that the impact of the actions specified in the national PA strategy (or an equivalent policy document) is monitored over the long term?

🞅 _____________________________________________________________

🞅 Don’t know

| Sample question 10 is derived from the following principle: *“Ministries of health have a crucial convening role* - bringing together other ministries needed for effective policy design and implementation”. [1] |
| --- |

1. Does the Ministry of Health (or an equivalent ministry) have a crucial convening role, that is, it brings together other ministries needed for the effective…

| … design of PA policies? | 🞅 Yes | 🞅 No | 🞅 Don’t know |
| --- | --- | --- | --- |
| … implementation of PA policies? | 🞅 Yes | 🞅 No | 🞅 Don’t know |

| Sample question 11 is derived from the following principle: *“Governments need to work together with* the private sector, health professional bodies, consumer groups, academics, the research community and other nongovernmental bodies if sustained progress is to occur”. [1] |
| --- |

1. When addressing the problem of insufficient PA, does the Government work together with…

| … the private sector? | 🞅 Yes | 🞅 No | 🞅 Don’t know |
| --- | --- | --- | --- |
| … health professional bodies? | 🞅 Yes | 🞅 No | 🞅 Don’t know |
| … consumer groups? | 🞅 Yes | 🞅 No | 🞅 Don’t know |
| … academics? | 🞅 Yes | 🞅 No | 🞅 Don’t know |
| … research community? | 🞅 Yes | 🞅 No | 🞅 Don’t know |
| … non-governmental bodies? | 🞅 Yes | 🞅 No | 🞅 Don’t know |

| Sample question 12 is derived from the following principle: “*A life-course perspective* on chronic disease prevention and control is critical. This starts with maternal and child health, nutrition and care practices, and carries through to school and workplace environments, access to preventive health and primary care, as well as community based care for the elderly and disabled people”. [1] |
| --- |

1. Is a *life-course perspective* integrated in the national efforts to address the problem of insufficient physical activity? *(A life-course perspective commences with maternal health, child health, and care practices, continues through to the school environment and later to the workplace environment, and includes the access to preventive health care, primary health care, and community-based care for disabled people and seniors)*

🞅 Yes

🞅 No

🞅 Don’t know

| Sample questions 13, 14 and 15 are derived from the following principles: “Strategies should explicitly address equality and diminish disparities; they should focus on the needs of the *poorest communities and population groups* - this requires a strong role for government. Furthermore, since women generally make decisions about household nutrition, strategies should be *gender* sensitive”. [1] |
| --- |

1. Does the national PA strategy (or an equivalent policy document) explicitly address equality and diminish disparities?

🞅 Yes

🞅 No

🞅 Don’t know

1. Does the national PA strategy (or an equivalent policy document) focus on the needs of the poorest communities and population groups?

🞅 Yes

🞅 No

🞅 Don’t know

1. Is the national PA strategy (or an equivalent policy document) *gender* sensitive?

🞅 Yes

🞅 No

🞅 Don’t know

| Sample question 16 is derived from the following principle: “Strategies need to draw substantially on existing *international standards* that provide a reference in international trade… WHO’s international leadership role in pushing forward the agenda on diet, physical activity and health is crucial”. [1] |
| --- |

1. Does the national PA strategy (or an equivalent policy document) draw substantially on existing *international standards* that provide a reference in international trade?

🞅 Yes

🞅 No

🞅 Don’t know
